# Should IQOS Emissions Be Considered as Smoke and Harmful to Health? A Review of the Chemical Evidence

**DOI:** 10.1021/acsomega.2c01527

**Published:** 2022-06-22

**Authors:** Clement N. Uguna, Colin E. Snape

**Affiliations:** University of Nottingham, Faculty of Engineering, Energy Technologies Building, Jubilee Campus, Triumph Road, Nottingham, NG7 2TU, U.K.

## Abstract

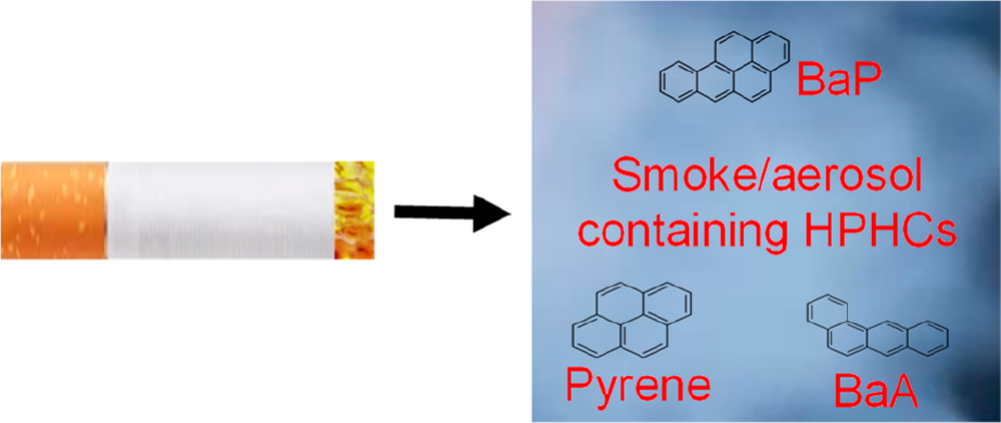

The chemical evidence that IQOS emissions fit the definition of both an aerosol and smoke, and that IQOS and potentially other heated tobacco products (HTPs) pose some harmful health threats from the range of compounds released even at somewhat lower concentrations is reviewed. Further, we address the yields of harmful and potentially harmful compounds (HPHCs), including polycyclic aromatic hydrocarbons (PAHs), and the constituents of IQOS emission that are diagnostic of pyrolysis to provide information on the temperatures reached in IQOS tobacco sticks. The HPHCs present in IQOS emissions are the same as in conventional cigarette smoke (CCs), analogous to emissions from earlier generation of HTPs classed as smoke. However, Philip Morris International (PMI) studies have to some degree underestimated IQOS aerosol HPHC yields, which are a factor of between 3.2 and 3.6 higher when expressed on a tobacco rather than an IQOS stick basis compared to the reference 3R4F cigarette. Further, IQOS emissions contain carbon particles, which fit definition of both aerosol and smoke. Continual reheating of deposited tar in the IQOS device will occur with real-life use, likely leading to generation of even higher concentrations of HPHCs and particulate matter. Despite IQOS not exceeding 350 °C, local hot spots could exist, causing formation of species (phenol/cresols, PAHs). It is recommended that the impact of repeated use to determine the levels of black carbon (insoluble organic matter) in the particulate matter, and the extent to which compounds in IQOS emissions are formed by pyrolysis need to be assessed rigorously. To address whether uneven temperature profiles in heat sticks can lead to potential hot spots that could, for example, lead to PAH formation, it is recommended that pyrolysis studies on tobacco and other constituents of HTPs are required in conjunction with more effort on heating tobacco blends under controlled temperature/time conditions.

## Introduction

It is well-established that conventional cigarette (CC) smoke is harmful to human health, contributing to the development of conditions such as lung cancer, respiratory disease such as chronic obstructive pulmonary disease (COPD), cardiovascular diseases and premature deaths,^1^ with second-hand smoke also being linked to adverse health effects.^1^ The accepted dangers of CC smoking have led to the emergence of both electronic (e-) cigarettes and heated tobacco products (HTP),^2^ including Glo manufactured by British American Tobacco (BAT) and the Tobacco Heating System (THS) or IQOS by Philip Morris International (PMI). E-cigarettes produce an aerosol from solutions containing a mixture of nicotine, glycerine, propylene glycol, water and flavouring chemicals depending on the different commercial brands.^3^ While, HTPs are electronic devices that heat a rod or stick containing cast tobacco sheet (IQOS) or reconstituted tobacco (Glo) made from ground tobacco powder prepared with ingredients such as glycerol, water, cellulose fiber, and guar gum to produce vapors.^4−6^ Hybrid devices, such as Japan Tobacco (JT) PLOOM TECH, generate nicotine aerosols by heating an e-liquid and passing the vapor through a capsule of tobacco.^7^

The claims by PMI, BAT, and other manufacturers that HTPs are less harmful than CCs are based, in part, that the devices being smoke free (generating a smokeless aerosol) and are heated to maximum temperatures of 350 °C for IQOS^6,8^ and 250 °C for Glo,^9^ compared to CCs reaching temperatures of 200–600 and 700–950 °C in the pyrolysis/distillation and combustion zones,^10^ respectively. Compared with CCs, a recent review of both industry and independent evidence covering 31 studies by Simonavicius et al.^11^ concluded that HTPs delivered up to 83% of the nicotine level while, overall, reducing harmful and potentially harmful toxicants and particulate matter by at least 62% and 75%, respectively. However, they also reported that generally higher concentrations of HPHCs were released in studies on humans compared to smoking devices.

PMI submitted a modified risk tobacco product (MRTP) application to the US Food and Drug Administration (FDA) in 2016 to market IQOS as a reduced risk alternative to CCs^12^ in the US. Since the launch of IQOS in some countries, there has been an ensuing debate on the extent to which IQOS is harmful to humans in relation to CCs. Indeed, according to Bialous and Glantz,^13^ PMI’s claim of IQOS being smoke free and beneficial to health was a means to bypass stringent tobacco regulation, making it easier to market IQOS, while gaining favorable taxation status in some countries around the world. While animal studies,^8,14−19^ and human clinical studies^20−23^ by PMI researchers claim that IQOS aerosol is significantly less harmful to human health than CC smoke, findings from independent reviews of PMI’s own data shows that IQOS aerosol is as harmful as CC smoke to human health.^24−26^ In addition, Davis et al.^27^ conducted similar cytotoxicity tests as Schaller et al.^8^ (a PMI study) and found that IQOS emissions are as harmful as CC smoke to human health both at high concentration for some cell types. Especially when exposed to more sensitive cells from human embryos (H9-Hesc) and respiratory systems (BEAS-2B) compared to the less sensitive cells (A549 cancer cell) or NIH/3T3 (mouse embryonic fibroblast) used by Schaller et al.^8^ From the independent human-based toxicological studies conducted, Jankowski et al.^28^ concluded that there is a potentially harmful impact of both active and passive HTP smoking on human health.

To complement the emerging toxicological evidence that IQOS and potentially other HTPs pose significant harmful health threats, this review considers the analytical evidence as to whether the HPHCs and other species present in HTP vapors can be considered as being smoke free and a lower risk than CC for human exposure. We focus on IQOS because it is the largest selling HTP globally and has been subjected to the most detailed study regarding the composition of the emissions released. Further, we address (i) the yields of HPHCs, including polycyclic aromatic hydrocarbons (PAHs) and phenols, as classes of carcinogens and cocarcinogens respectively, which were not addressed in any detail in the review by Simonavicius et al.,^11^ (ii) the constituents of IQOS emission that are diagnostic of pyrolysis in relation to those generated by tobacco and, more generally, biomass, and provide information on the temperatures reached, and (iii) compounds present in higher concentrations in IQOS emissions compared to CC smoke.

A key issue for all these questions is the maximum temperatures reached in IQOS, where Cozzani et al.^4^ (a PMI funded study) measured the maximum temperature of IQOS tobacco substrate to be 320 °C (accuracy ±2.5 °C) using a 0.25 mm diameter thermocouple inserted into the tobacco substrate via a 0.5 mm diameter hole drilled into the side of the outer casing of IQOS holder. However, the independent study by Auer et al.^29^ quoted the temperature of IQOS device to be 330 °C and other PMI studies identified temperatures as high as 350 °C for IQOS heater blade.^6,8^ We also consider the evidence for IQOS generating main and side stream emissions that potentially makes the device unsuitable for indoor environment. Finally, we recommend analytical protocols that will resolve many of the uncertainties identified regarding the extent to which pyrolytic decomposition, which controls both the concentrations of individual species and the overall tar yields, is occurring. Resolving these uncertainties, together with more independent toxicological data will better inform future regulation of IQOS and other HTPs.

## To What Extent Can IQOS Be Considered Smoke Free?

To ascertain the extent to which IQOS can be considered as smoke free, we first summarize the mechanism of generation of IQOS aerosol and that of CC smoke before considering the various definitions for smoke. According to Baker and Bishop,^10^ the interior burning zone of CC can be divided into two regions, an exothermic combustion zone and an endothermic pyrolysis/distillation zone. As air is drawn into the cigarette during a puff, oxygen is consumed by combustion in the exothermic combustion zone, which releases heat of between 700 and 950 °C. At the pyrolysis/distillation zone that is low in oxygen levels the temperatures are approximately between 200 and 600 °C, and majority of the smoke products are formed in this region via endothermic mechanism.^10^ A highly concentrated and probably supersaturated vapor generated in the pyrolysis/distillation zone drawn down the tobacco rod during a puff forms the mainstream smoke.^10^ As the generated vapor is drawn out of the pyrolysis/distillation region during a puff, it cools very rapidly in the presence of diluting air entering at the paper burn line. This brings the vapors of the less volatile compounds quickly to their saturation point and condensation occurs as the vapor cools below about 350 °C, resulting to the formation of a dense aerosol consisting of growing droplet particles.^10^ Thus, CC smoke is formed by endothermic mechanisms via pyrolytic distillation of tobacco by condensed vapor drawn from the pyrolysis/distillation zone down the tobacco rod at lower temperature during puffing, independent of the self-sustained combustion process in the exothermic combustion zone at higher temperature (700–950 °C). Implying that smoke can also be generated by evaporation/distillation of organic compounds by simply the application of heat in the absence of combustion.

As reported by Cozzani et el,^4^ IQOS aerosol is generated by the same endothermic process as CC smoke, at temperatures up to 320 °C. IQOS tobacco temperatures have been reported to be up to 320 °C measured by Cozzani et al.,^4^ 330 and 350 °C reported by Auer et al.,^29^ and other PMI studies^6,8^ respectively, which is high enough to suggest pyrolysis is a major contributor to IQOS aerosol generation. Further evidence is provided by the high tar yields from IQOS compared to CC in a later section. The fact that much of the IQOS aerosol is generated via endothermic pyrolytic reactions by an endothermic process as for CC smoke suggests that IQOS aerosol can also be classed as smoke generated at lower temperature as proposed previously by Auer et al.^29^

Table 1 lists some of the various definitions that have been used to define smoke and aerosols, with respect to CC and HTPs. Aerosols encompass all suspensions of solid or liquid particles in a gas. For example, CORESTA, an industry association in which every major tobacco company is a member,^2^ defines aerosol as being a suspension of particles (comprising only liquids or a mixture of liquids and solids) in air or gas^2^ (Table 1). PMI^30^ has defined smoke as an aerosol containing liquid and solid particles (particulate matter) formed from combustion and high temperature pyrolysis. On the other hand, PMI^30,31^ defines IQOS aerosol as being formed at lower temperatures (320 °C) from drying, evaporation, and thermochemical decomposition (torrefaction/low temperature pyrolysis) of tobacco with no solid particles being generated (Table 1). This contrasts to the PMI study by Meisutovic-Akhtarieva et al.,^32^ which indicated that IQOS emissions contain PM2.5s, and Ruprecht et al.^3^ (an independent study) that confirms the presence of PM1, PM2.5, and PM10s, as well as black carbon detected in the 370 UV nm range in IQOS emission in concentrations lower than CC smoke. PM1, PM2.5, and PM10s may or may not contain solid particles, while black carbon is a generic term covering all solid carbonaceous material and has been attributed to PAHs and other organic compounds, such as are found in wood smoke, biomass-burning smoke, and tobacco smoke.^33,34^ It has also been estimated that approximately 83% of combustible cigarette smoke in gaseous form is invisible^35^ (Table 1). However, importantly, it includes carbonaceous material not extractable in common solvents, and this insoluble carbon fraction is the major component arising from incomplete combustion.

**Table 1:** Some Definitions and Descriptions of Smoke and Aerosols with Respect to Conventional Cigarettes and HTPs

definition/description	affiliation
an aerosol is suspension of particles in air or gas; the particles can be composed of only liquids or a mixture of liquids and solids^2^	tobacco industry (accessed 1 March 2022)
cigarette smoke aerosol is a complex and dynamic mixture of gases, liquid droplets, and solid particles suspended in air; generated by combustion, pyrolysis, and pyrosynthesis processes that overlap with low temperature distillation and sublimation processes^4^	tobacco industry (published 2020)
IQOS aerosol contains low levels of low molecular weight gases (such as CO, CO_2_ and NH_3_), aldehydes, ketones, low molecular weight hydrocarbons, and aromatics formed from drying, evaporation, and thermochemical decomposition (torrefaction/low temperature pyrolysis) of tobacco^4,30^	tobacco industry (published 2020,^4^ accessed 1 March 2022^30^)
combustible cigarette smoke consists of an aerosol containing liquid droplets (particulate phase) suspended in a carrier gas and surrounded by its own gas vapor phase^8^	tobacco industry (published 2016)
an aerosol is a suspension of solid or liquid particles in a gas, usually air^31^	tobacco industry (accessed 17 November 2020)
smoke is an aerosol that contains solid particles and thousands of chemicals that are generated at high temperatures when a material combusts^31^	tobacco industry (accessed 17 November 2020)
smoke released by IQOS was described to contain elements from pyrolysis and thermogenic degradation that are the same harmful constituents of conventional tobacco cigarette^29^	independent study (published 2017)
it has been estimated that approximately 83% of combustible cigarette smoke is in a gaseous form that is not visible^35^	independent study (published 2013)
emissions from early generation heated tobacco product (Eclipse) were classed as smoke^39^	tobacco industry (published 2004)

Auer et al.^29^ proposed that IQOS emissions should be classed as smoke as they contain compounds from pyrolysis and thermogenic degradation that are the same HPHCs as for conventional tobacco cigarette (Table 1). PMI responded^36^ despite the yields of PAHs and other HPHCs already reported by PMI^37,38^ being higher than or within the range reported by Auer et al.,^29^ except for acenaphthene (Table 3). The lower yields of most HPHCs observed in IQOS smoke by Auer et al.^29^ might be due to the less intense International Organization for Standardization (ISO) regimen used for generating the smoke.

It is also important to note that emissions from earlier generation of HTPs (e.g., Eclipse) were classed as smoke by a study affiliated to the manufacturer^39^ (Table 1), and the emissions were also found to contain soot (black carbon).^40^ In addition, Vivarelli et al.^41^ refers to IQOS aerosol as smoke containing carcinogenic compounds, including aldehydes and polycyclic aromatic hydrocarbons, that are sign of incomplete combustion and degradation of tobacco. This raises the question as to what extent IQOS emissions differ from those from these earlier HTPs so as not to be classed as smoke. Thus, smoke can be considered a class of aerosol within these all-encompassing definitions and description and those presented in Table 1. Pyrolysis rather than evaporation contributes to the bulk of HPHCs and other species released from both IQOS and earlier HTPs as they do not exist as such in tobacco. The crux of the argument is, therefore, does the release of particulate matter containing HPHCs (and possibly insoluble black carbon) formed by extensive thermal decomposition, although at much lower temperatures and not undergoing partial combustion as for CC smoke, classify IQOS emissions as smoke? To put the argument succinctly “can smoke exist without fire?”

## Basis of Comparisons between IQOS and CC

Before discussing the yields of tar, HPHCs and other species between IQOS and CC, it is important to address the basis of the comparisons made. All comparisons to date made compare an IQOS stick with the 3R4F reference cigarette. However, a mass of tobacco basis is also useful to provide a “like against like” comparison to understand differences in the formation and release of specific HPHCs. Our calculation detailed in Table 2 using tobacco content of 3R4F cigarette^42,43^ indicates that, on a tobacco basis, the yields of HPHCs and other constituents in IQOS aerosol need to be multiplied by a factor of between 3.2 and 3.6 to provide a comparison on a tobacco basis. An IQOS tobacco stick contains between 177.2 and 203.3 mg tobacco (depending on brand) as revealed from product ingredient information on PMI Web site,^44^ and as shown in Table 2 PMI studies^4,8^ compared IQOS aerosol constituents to that of 3R4F smoke generated from 645.5 mg of tobacco.

**Table 2:** Calculation of the Mass of 3R4F Cigarette Tobacco Smoked to Generate Smoke in Two PMI Studies (Cozzani et al.^4^ and Schaller et al.^8^)

tobacco content of the 3R4F reference cigarette reported	753 mg^42^ and 760 mg^43^
entire length of 3R4F cigarette together with its filter	83.9 mm^42^
3R4F cigarette filter length	26.7 mm^42^
3R4F cigarette rod length holding 753 mg of tobacco	83.9 mm −26.7 mm = 57.2 mm
amount of tobacco contained in 1 mm of rod length assuming 57.2 mm rod length contain 753 mg of tobacco parked uniformly over its length	[(753 mg × 1 mm)/57.2 mm = 13.2 mg]
butt length of 3R4F cigarette smoked to generate smoke (Cozzani et al.^4^ and Schaller et al.^8^)	35 mm
3R4F tobacco length burnt to generate smoke by smoking 3R4F cigarette to a butt length of 35 mm (Cozzani et al.^4^ and Schaller et al.^8^)	filter plus unburnt tobacco minus butt length of 35 mm after smoking (83.9 mm – 35 mm = 48.9 mm)
amount of 3R4F cigarette tobacco smoked to generate smoke (Cozzani et al.^4^ and Schaller et al.^8^)	tobacco contained in 1 mm length of 3R4F rod times length of the tobacco rod burnt divided by 1 mm rod length of 3R4F cigarette [(13.2 mg × 48.9 mm)/1 mm = 645.5 mg]

## Tar, Nicotine, and Particulate Matter Yields

**Figure 1:**
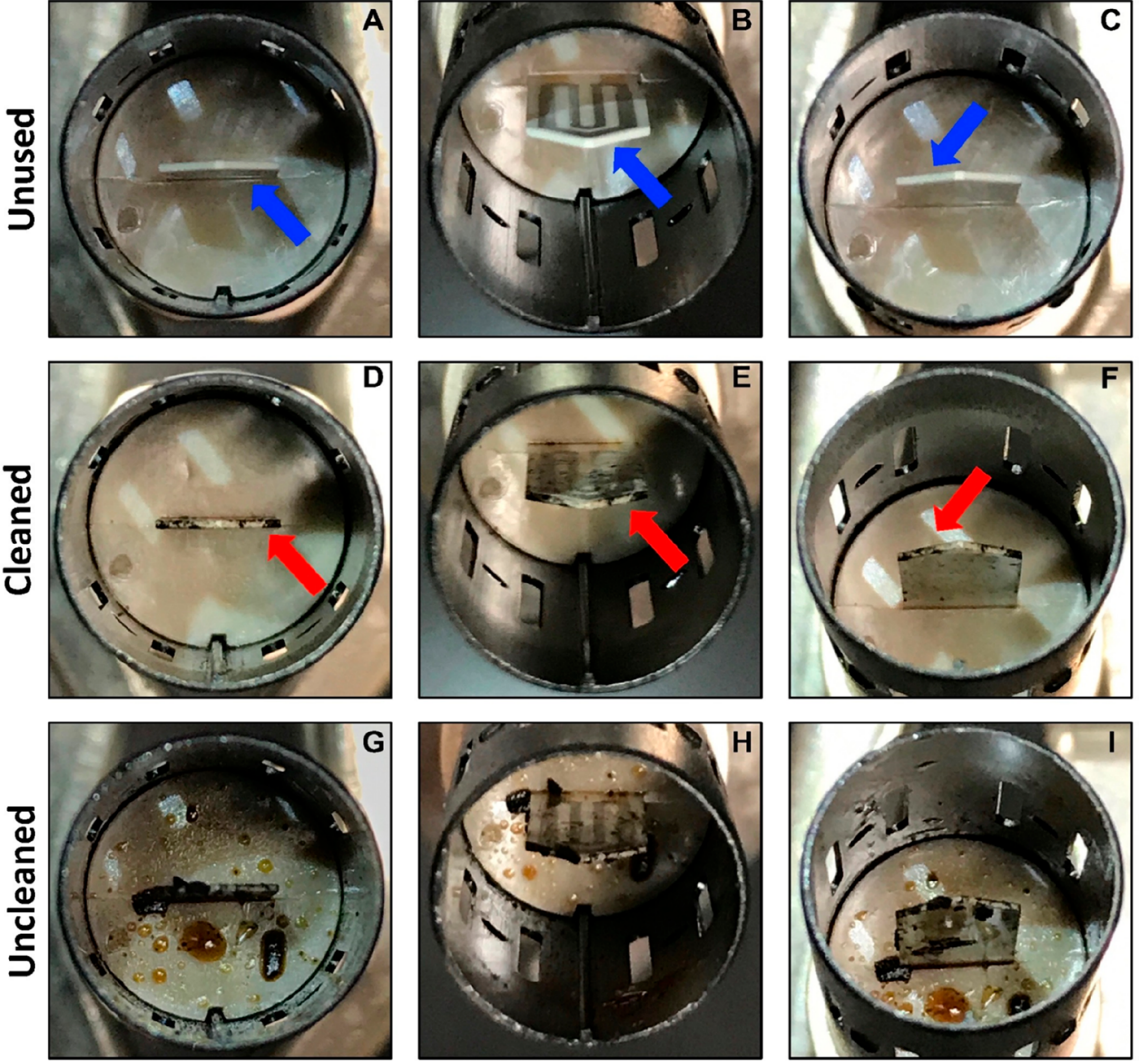
Internal view of the IQOS holder. (A–C) Clean, unused holder showing heater (blue arrows). (D–F) Used holder that was cleaned after every use; black residue remains on heater (red arrows). (G–I) Used holder that was not cleaned between uses (10 uses). Adapted from an independent study Davis et al.^45^ and reproduced with the permission of BMJ Publishing Group, Ltd.

Davis et al.^45^ evaluated the performance of the IQOS device and confirmed the deposits of a brown liquid (tar) on the holder and a black residue on the heater, the latter attributed to charring, after use of several heat sticks (Figure 1). Charring would be confirmed by demonstrating the residue contained a high proportion of insoluble material (black carbon). Following PMI’s recommendation of cleaning the device after using 20 heatsticks,^45^ the continual reheating of the deposited tar and char is likely to result in the generation of higher concentrations of PAH and other HPHCs than from single use, which is the only data reported thus far by Davis et al.^45^ Consistent with the study of McGrath et al.^46^ that showed that the yield of PAH increased by reheating char initially obtained from pyrolysis of tobacco at 350 °C for 10 min and at 600 °C for 10 min. PAH concentrations in IQOS vapor will increase with continual reheating of the deposited tar and char at the operational temperature of IQOS device because polycyclic aromatic structures are known to exist in the residual solids of tobacco and tobacco components at temperatures as low as 300 °C,^46^ with longer times compensating for lower temperatures during pyrolysis. Therefore, analysis of the condensable and vapor-phase species released from IQOS after repeated use is essential to gain a fuller appreciation of the HPHCs and particulate matter formed. Indeed, the identification of insoluble black carbon would prove that extensive charring has occurred. Jankowski et al.^28^ also highlighted that most of the research regarding the chemical composition was carried out on brand new devices and overall emission levels could be higher for used devices, as indicated from the study by Davis et al.^45^ (Figure 1).

**Figure 2:**
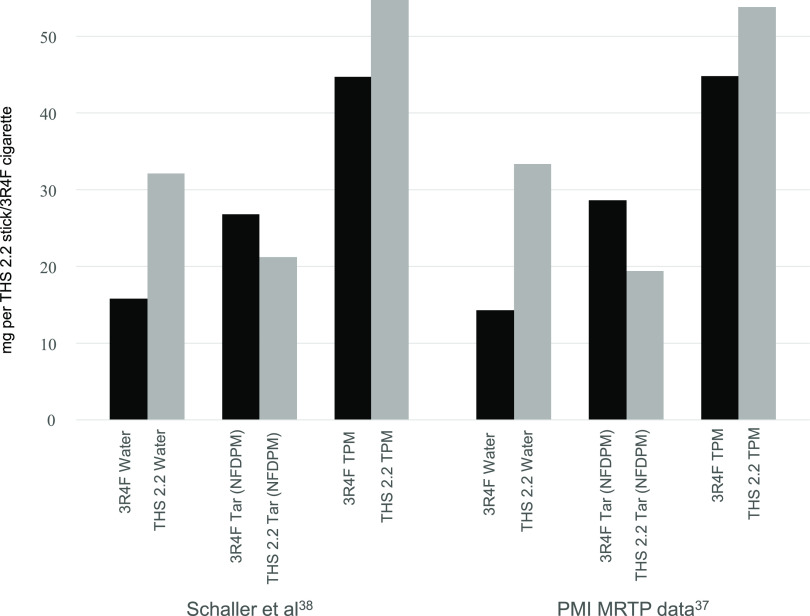
Comparison of IQOS (THS 2.2) aerosol total particulate matter (TPM), tar (NFDPM), and water yields to 3R4F reference cigarette smoke yields generated under HCI regimen. TPM does not include compounds in the gas vapor phase (GVP). However, on an equivalent tobacco basis, the IQOS TPM and NFDPM yields should be multiplied by at least 3.2. Note: 3R4F used for each study is the same sample; any difference in yield reflects experimental error from different experiments. PMI MRTP data^37^ are average yields of regular and menthol heatsticks, while Schaller et al.^38^ is for FR1 blend.

Tar, also referred to nicotine free dry particulate matter (NFDPM), is present in appreciable amounts in HTP emissions,^4,5,38^ a ubiquitous overall product of both low and high temperature pyrolysis of tobacco and biomass.^46−49^ Unless the tar has been extracted, it will also include any insoluble black carbon that may be present. Figure 2 compares the total particulate (TPM, tar plus nicotine plus water), tar (NFDPM), and water yields collected from IQOS emission and 3R4F reference cigarette smoke under the Health Canada Intense (HCI) smoking regimen. The IQOS aerosol TPM yield is higher than that for 3R4F smoke because of the water yield being higher resulting from the much higher water content of the tobacco stick^4,6^ and the IQOS emission tar yield is on average 73% (Figure 2) and nicotine yield 64% (Table 3) of the amount in 3R4F reference cigarette smoke. However, on a tobacco basis, the tar and nicotine yields are roughly twice as high from IQOS, consistent with the higher temperatures in CC resulting in much of the primary tar and nicotine being cracked. Indeed, White et al.^47^ during pyrolysis of tobacco between 250 and 550 °C under the more intense Federal Trade Commission (FTC) method (50 cc puff volume, 2 s duration and 30 s interval), observed maximum tar yield at 475 °C, after which tar yield reduced at 550 °C to about the same value as obtained at 325 °C. In the study by White et al.^47^ maximum nicotine yield was observed at 250 °C (0.83 mg per tobacco tablet) and the yield reduced by a factor of 3.6 at 550 °C (0.23 mg per tobacco tablet). This is because of the thermal breakdown of the tar and nicotine to lower molecular weight organic compounds at higher pyrolysis temperature. Similar thermal breakdown of tar and nicotine will occur for the 3R4F cigarette with temperatures of up to 600 °C in the pyrolysis/distillation zone,^10^ further explaining why IQOS tar and nicotine yields in Figure 2 and Table 3 would be higher than for the 3R4F cigarette on an equivalent tobacco basis.

**Table 3:** Comparison of Constituent Released from an IQOS (THS 2.2) Stick Vapors with Those from the 3R4F Reference Cigarette Smoke^a^

	Cozzani et al.^4^	PMI MRTP data^37^	Schaller et al.^38^	Auer et al.^29^	
constituents detected	amount per heatstick (HCI regimen)	amount per heatstick (HCI regimen)	amount per heatstick (HCI regimen)	amount per heatstick (ISO regimen)	range of values obtained per 3R4F cigarette^4,37,38^
carbonyls					
acetaldehyde^c^ (μg)	211–230 (13–14)^b^	197.2–199.4 (12–12)^b^	181–267 (11–16)^b^	133	1656–1713
acetone^c^ (μg)	31–35.9 (4–5)^b^	31.5–32.5 (5)^b^	28.7–41.9 (4–6)^b^	0.9	685–708
butyraldehyde (μg)	22.5–23.1 (25)^b^		15.3–25.6 (18–31)^b^		83.5–91
acrolein^c^ (μg)	8.4–10.7 (5–7)^b^	9.20–9.36 (5)^b^	5.83–14.17 (4–9)^b^		161–177
crotonaldehyde^c^ (μg)	0.988–3.292 (2–6)^b^	3.29 (6)^b^	3.29 (6)^b^	0.7	51.7–55.2
formaldehyde^c^ (μg)	6.1–9.1 (7–10)^b^	7.10–7.68 (10–11)^b^	4.58–13.42 (5–15)^b^	3.2	70.2–88.9
methyl ethyl ketone^c^ (μg)	7.0–7.6 (4)^b^	7.08–7.10 (4)^b^	6.42–10.15 (4–6)^b^		183–197
propionaldehyde^c^ (μg)	13.7–14.9 (11–12)^b^	12.2–12.4 (10)^b^	12.3–15.2 (10–12)^b^	7.8	122–125
vinyl acetate^c^ (ng)		60.1–66.4 (9–10)^b^			646
glycols					
glycerin (mg)	4.38–4.39 (190)^b^		3.72–5.69 (163–250)^b^		2.28–2.30
hydrocarbons					
1,3-butadiene^c^ (μg)	0.3 (0.3)^b^	0.230–0.273 (0.2–0.3)^b^	0.095–0.347 (0.1–0.4)^b^		93–98.2
benzene^c^ (μg)	0.5–0.6 (0.6–0.7)^b^	0.483–0.561 (0.6–0.7)^b^	0.442–1.010 (0.5–1.0)^b^		81.1–90.7
ethylbenzene^c^ (μg)		0.132–0.151 (0.9–1.0)^b^			14.8
hydroquinone (μg)	7.0–7.4 (8)^b^		4.77–9.39 (5–11)^b^		88.3–92.5
toluene^c^ (μg)	1.9–2.0 (1)^b^	1.40–1.65 (1)^b^	1.77–3.05 (1–2)^b^		137–158
styrene^c^ (μg)	0.7–0.8 (4)^b^	0.328–0.336 (3)^b^	0.468–1.128 (3–7)^b^		13–18.2
isoprene^c^ (μg)	2.3–2.6 (0.3)^b^	1.33–1.62 (0.2)^b^	1.01–4.34 (0.1–0.5)^b^		812–913
metals					
arsenic^c^ (ng)		1.20 (15)^b^	1.20–1.43 (15–18)^b^		7.99–8.23
cadmium^c^ (ng)		0.09–0.28 (0.1–0.3)^b^	0.280 (0.3)^b^		94–99.4
lead^c^ (ng)			1.62–3.80 (5–12)^b^		31.9
mercury^c^ (ng)		1.88–2.11 (43–48)^b^	0.70–1.60 (15–34)^b^		4.36–4.67
nitrogen-containing compounds					
acetamide^c^ (μg)		3.21–3.28 (26–27)^b^	2.24–6.28 (17–48)^b^		12.3–13.0
acrylamide^c^ (μg)		1.64–1.80 (38–42)^b^	0.78–3.56 (17–79)^b^		4.33–4.5
acrylonitrile^c^ (μg)	0.2 (0.8)^b^	0.107–0.112 (0.5)^b^	0.107–0.335 (0.4–1.0)^b^		22.5–26.1
ammonia^c^ (μg)		13.14–13.38 (41–42)^b^	5.3–97.2 (17–312)^b^		31.2–31.7
hydrogen cyanide^c^ (μg)		2.06–2.17 (0.5)^b^	4.37–10.07 (1–3)^b^		364–433
3-aminobiphenyl (ng)	0.012 (0.3)^b^		0.004–0.014 (0.1–0.3)^b^		4.09–4.5
4-aminobiphenyl^c^ (ng)	0.016 (0.5)^b^	0.008–0.010 (0.3–0.4)^b^	0.005–0.028 (0.2–1.0)^b^		2.77–3.10
1-aminonaphthalene^c^ (ng)	0.07 (0.3)^b^	0.027 (0.1)^b^	0.027–0.091 (0.1–0.4)^b^		18.4–22.4
2,6-dimethlyaniline^c^ (ng)		0.270–0.316 (3–4)^b^			8.01
2-aminonaphthalene^c^ (ng)	0.04 (0.3)^b^	0.012 (0.1)^b^	0.012–0.056 (0.1–0.3)^b^		11.6–16.2
nicotine^c^ (mg)	1.37–1.38 (69)^b^	1.23 (66)^b^	0.62–1.64 (33–87)^b^		1.87–2.0
nitromethane^c^ (ng)		44.3–51.2 (5–6)^b^			809
*o*-anisidine^c^ (ng)		0.124–0.131 (2–3)^b^			5.20
*o*-toluidine^c^ (ng)		1.08 (1)^b^	0.542–3.094 (0.5–3.0)^b^		103.9–105
quinoline^c^ (μg)	0.003–0.011 (0.6–2)^b^	0.011 (3)^b^	0.011 (3)^b^		0.409–0.49
pyridine (μg)	7.4–7.8 (21–22)^b^		5.53–11.18 (18–35)^b^		31.5–35.1
2-nitropropane^c^ (ng)		6.0–8.40 (16–23)^b^			36.5
other constituents					
Tar (NFDPM) (mg)	16.5–17.9 (60–65)^b^	18.7–20 (65–70)^b^	10.6–25.5 (40–95)^b^		26.8–28.6
TPM (mg)	54–55.2 (118–120)^b^	52.8–54.8 (118–122)^b^	46.8–57.8 (105–129)^b^		44.7–45.8
vinyl chloride^c^ (ng)		0.657 (0.5)^b^	2.19–3.92 (2–4)^b^		100.8–128
water (mg)	34.7–37.3 (213–229)^b^	32.9–33.6 (230–235)^b^	25.6–40.9 (162–258)^b^		14.3–16.3
oxygenated compounds					
carbon monoxide^c^ (mg)	0.159–0.54 (0.5–2)^b^	0.067(0.2)^b^	0.223–0.567(0.7–2)^b^		30.6–33.4
ethylene oxide^c^ (μg)		0.198–0.234 (0.9–1)^b^	0.119–0.324 (0.5–1)^b^		21.2–24.10
benzo[*b*]furan^c^ (μg)		0.027–0.030 (5)^b^			0.592
furan^c^ (μg)		4.43–4.49 (8)^b^			58.3
nitric oxide (μg)			3.7–51.4 (0.7–10)^b^		510
nitrogen oxide (μg)			4.2–51.4 (0.7–9)^b^		571
propylene oxide^c^ (ng)		158–159 (17)^b^	65–109 (6–10)^b^		930–1110
PAHs					
naphthalene^c^ (ng)		5.94–7.34 (0.5–0.6)^b^		1.6	1197
1-methylnaphthalene (ng)		6.78–8.36 (0.7–0.8)^b^			1016
2-methylnaphthalene (ng)		29.8–35.3 (3–4)^b^			953
acenaphthylene (ng)		2.44–2.97 (1–2)^b^		1.9	196
acenaphthene (ng)		0.683–0.702 (0.5)^b^		145	129
anthracene (ng)		0.786–0.942 (0.7–0.8)^b^		0.3	120
fluorene (ng)		8.1–10.3 (2–3)^b^		1.5	409
phenanthrene (ng)		5.34–6.62 (3)^b^		2.0	201
benz[*a*]anthracene^c^ (ng)		2.01–2.75 (6–9)^b^	0.36–20.52 (1–75)^b^	1.8	27.2–31.6
chrysene^c^ (ng)		2.93–3.86 (7–9)^b^		1.5	40.7
fluoranthene (ng)		7.6–10.5 (7–10)^b^		7.3	107
pyrene (ng)		8.4–11.4 (9–13)^b^	1.97–74.09 (2–93)^b^	6.4	79.3–88.9
benzo[*b*]fluoranthene^c^ (ng)		0.84–1.20 (6–9)^b^		0.5	13.9
benzo[*k*]fluoranthene^c^ (ng)		0.395–0.607 (8–12)^b^		0.4	4.86
benzo[*j*]fluoranthene (ng)		0.574–0.849 (8–12)^b^			7.30
benzo[*c*]phenanthrene^c^ (ng)		0.86–1.29 (11–16)^b^			7.96
benzo[*j*]aceanthrylene^c^ (ng)		0.104 (9)^b^			1.15
benzo[*a*]pyrene^c^ (ng)	0.60–0.61 (3–4)^b^	0.74–1.12 (5–7)^b^	0.35–4.46 (2–30)^b^	0.8	15.0–17.3
perylene (ng)		0.379 (10)^b^			3.78
benzo[*e*]pyrene^c^ (ng)		0.496–0.680 (8–10)^b^			6.54
benzo[*g*,*h*,*i*]perylene (ng)		0.337 (12)^b^			2.85
cyclopenta[c,d]pyrene^c^ (ng)		1.12–1.96 (19–33)^b^			6.0
dibenzo[*a*,*h*]anthracene^c^ (ng)		0.124 (16)^b^	0.413 (52)^b^		0.79–0.797
indeno[1,2,3-cd]pyrene^c^ (ng)		0.337 (6)^b^			5.36
phenols					
catechol^c^ (μg)	14.3–14.7 (17)^b^	12.7–12.9 (13)^b^	10.6–16.3 (12–18)^b^		84.2–98.1
*m*-cresol^c^ (μg)	0.03 (1)^b^	0.030–0.033 (1)^b^	0.019–0.116 (1–3)^b^		3.2–3.61
*o*-cresol^c^ (μg)	0.06–0.07 (2)^b^	0.041–0.42 (1)^b^	0.041–0.113 (1–3)^b^		3.76–4.11
*p*-cresol^c^ (μg)	0.07 (0.9)^b^	0.034–0.040 (0.5–0.6)^b^	0.034–0.122 (0.4–1.0)^b^		6.56–8.86
phenol^c^ (μg)	1.3–1.4 (10–11)^b^	0.812–0.941 (6–7)^b^	0.72–1.59 (5–11)^b^		12.8–14.4
resorcinol (μg)	0.016–0.055 (0.8–3)^b^		0.055–0.080 (3–5)^b^		1.75–2.0
TSNAs					
4-(methylnitrosamino)-1-(3-pyridyl)-1-butanone (NNK)^c^ (ng)		6.92–9.00 (3–4)^b^	2.0–29.3 (0.8–11)^b^		232–261
*N*-nitrosonornicotine (NNN)^c^ (ng)		9.5–15.2 (3–5)^b^	3.0–57.1 (1–20)^b^		277–284
*N*-nitrosoanabasine (NAB) (ng)			0.77–8.89 (3–29)^b^		30.3
*N*-nitrosoanatabine (NAT) (ng)			4.9–63.9 (2–24)^b^		269

a The results compare the yields from an IQOS stick with the reference 3R4F cigarette; however, on an equivalent tobacco basis, the IQOS yields should be multiplied by at least 3.2. HCI, Health Canada Intense smoking regimen; ISO, International Organization for Standardization smoking regimen; PAHs, polycyclic aromatic hydrocarbons; TSNAs, tobacco-specific nitrosamines. Note: For consistency with other data in the table, only yields reported for THS 2.2 by Auer et al.^29^ are presented here because the authors reported yields of reference cigarette that were different from 3R4F reported by the other studies.

b Compound in IQOS aerosol as a percentage of amount in 3R4F reference cigarette smoke.

c Compound in FDA list of HPHCs.

## HPHC Yields

Table 3 compares the yields of HPHCs in IQOS (THS 2.2) emission to amounts in 3R4F reference cigarette smoke from 3 PMI studies (Cozzani et al.,^4^ Schaller et al.^38^ and data used by PMI in their US MRTP application^37^), as well as presenting HPHCs in IQOS (THS 2.2) aerosol from an independent study (Auer et al.^29^). The yields are compared on the basis of a 3R4F cigarette against an IQOS heat stick as opposed to a given quantity of tobacco. As already discussed, the tobacco in IQOS heat stick is 3.2 to 3.6 times less than the 3R4F tobacco smoked to generate its smoke for comparison. These compounds include carbonyls and other oxygenated compounds including phenols, nitrogen-containing compounds, such as pyridine and aromatic amines and hydrocarbons, including polycyclic aromatic hydrocarbons (PAHs). However, the data presented in Table 3 are only for constituents where reductions in yield were observed compared to the 3R4F reference cigarette. Consequently, Table 3 reflects the conclusion drawn by Schaller et al.^8^ that PAHs, aromatic amines, phenols, and aldehydes are generally 75% lower and the majority of the HPHCs are 90% lower in IQOS aerosol compared to CC smoke for the 3R4F reference cigarette. However, the situation is complicated because PAHs and other HPHCs are introduced into tobacco during curing,^50,51^ and the background PAH levels in IQOS are addressed later. Regarding heavy metals, with the notable exception of Hg, reduction factors are over 75%, while the yield of carbon monoxide (a marker of combustion) in IQOS emission is between 0.2% and 2% of the amounts in 3R4F reference cigarette smoke (Table 3). The lower yield of carbon monoxide in IQOS aerosol compared to 3R4F smoke is due to partial combustion being avoided, although the carbon monoxide yield would be at least between 0.6% and 6% on an equivalent tobacco basis as 3R4F tobacco smoked.

The conclusion reached by Schaller et al.^8^ is considered to result from comparing aerosol constituents from IQOS tobacco stick containing between 3.2 and 3.6 times less tobacco than amount used to generate 3R4F cigarette smoke for comparison. On an equivalent tobacco basis as 3R4F tobacco, the overall reduction in yields of PAHs, aromatic amines, phenols, and aldehydes as concluded by Schaller et al.^8^ would be at least 20% lower and the majority of the HPHCs 68% lower in IQOS aerosol compared to CC smoke for the 3R4F reference cigarette. It is important to note that our estimate of IQOS aerosol HPHCs been underestimated by a factor of between 3.2 and 3.6 was based on the assumption that the entire 177.2 mg or 203.3 mg of IQOS tobacco was consumed during the aerosol generation. The underestimation might be higher if the entire IQOS tobacco was not consumed during the aerosol generation.

## Compounds Present in IQOS Emissions in Higher Concentrations than CC Smoke

Going beyond the lower yields of the known HPHCs, significantly higher yields of compounds not in the FDA list of toxicants in IQOS aerosol compared to the 3R4F reference cigarette smoke have been reported.^26,52^ When the FDA reviewed PMI’s data, across the three IQOS variants, there were 80 constituents exclusive or higher in IQOS than CCs. Of these, 4 were possible or probable human carcinogens, 19 generated alerts in the OECD QSAR toolbox, and 9 were identified by PMI to be of toxicological concern.^53^ To illustrate this point further, Table 4 presents the yields of these compounds from a PMI study,^52^ and PMI MRTP application data reported by an independent study.^26^ These are predominately oxygen-containing compounds including acids, aldehydes, ketones, and furans but also some nitrogen-containing compounds, including pyrole, pyridine, and quinoline species. Of these, 22 were at least 200% higher and seven at least 1000% higher than in 3R4F reference cigarette smoke^26^ (Table 4) and would even be much higher on an equivalent tobacco basis, as already discussed for HPHCs. The potential health effects of these compounds are not yet known, but they may act in tandem with other species in the same way that phenols can enhance the carcinogenicity of PAHs generated from IQOS pose to users. Regarding a known health effect, Davis et al.^45^ observed the release of formaldehyde cyanohydrin from the IQOS polymer filter, that is metabolized in the liver and broken down into formaldehyde and cyanide.^45^

**Table 4:** Yields of Constituents Not in the FDA List of HPHCs that Are Higher in IQOS Aerosol than in 3R4F Smoke under the HCI Regimen^a^

	St. Helen et al.^26^	Bentley et al.^52^	
constituents detected	amount per THS 2.2 heatstick	amount per THS 2.2 heatstick	range of values obtained per 3R4F cigarette^26,52^
acids			
benzoic acid, 2,5-dihydroxy-methyl (μg)	4.55 (209)^b^		2.18
3-methylvaleric acid (μg)	5.1 (140)^b^		3.63
3-methylpentanoic acid (μg)		14.5 (113)^b^	12.8
carbonyls			
ethyl linoleate (μg)	0.135 (1688)^b^		0.008
ethyl linolenate (μg)	0.614 (401)^b^		0.153
2-furancarboxaldehyde,5-methyl (μg)	11.1 (378)^b^		2.94
ethyl dodecanoate (ethyl laurate) (μg)	0.023		not detected
hexadecanoic acid, ethyl ester (μg)	0.491 (6138)^b^	6.43 (6430)^b^	0.008–0.1
phenylacetaldehyde (μg)	1.41 (267)^b^		0.529
stearate, ethyl (μg)	0.074 (2467)^b^		0.003
glycols			
propylene glycol (μg)	175 (738)^b^	643 (717)^b^	23.7–89.6
hydrocarbons			
heptacosane (μg)		10.2 (121)^b^	8.41
benzene, 1,2,3,4-tetramethyl-4-(1-methylethenyl) (μg)	0.006 (120)^b^		0.005
butylated hydroxytoluene (μg)	0.132 (1886)^b^		0.007
eicosane, 2-methyl (μg)	0.05 (357)^b^		0.014
heneicosane, 2-methyl (μg)	0.063 (300)^b^		0.021
nitrogen compounds			
2-formyl-1-methylpyrrole (μg)	0.128 (200)^b^		0.064
4(*H*)-pyridine, *N*-acetyl (μg)	0.296 (264)^b^		0.112
isoquinoline, 3-methyl (μg)	6.29 (126)^b^		4.99
maltoxazine (μg)	0.077 (203)^b^		0.038
pyridoxin (μg)	0.699 (133)^b^		0.526
other constituents			
1,2-propanediol, 3-chloro (μg)	9.94 (168)^b^		5.93
1*H*-indene, 2,3-dihydro-1,1,5,6-tetramethyl (μg)	0.026 (186)^b^		0.014
3-chloro-1,2-propanediol (μg)		16.1 (196)^b^	8.21
*cis*-sesquisabinene hydrate (μg)	0.061		not detected
ergosterol (μg)	3.18 (201)^b^		1.58
labdane-8,15-diol, (13S) (μg)	0.143 (953)^b^		0.015
lanost-8-en-3-ol, 24-methylene-, (3beta) (μg)	6.3 (391)^b^		1.61
α-cembratriene-diol (μg)		8.49 (2160)^b^	0.393
*p*-menthan-3-ol (μg)	0.786 (244)^b^		0.322
other oxygenated compounds			
furfural (μg)	31.1 (120)^b^	47.4 (124)^b^	25.9–38.3
*trans*-4-hydroxymethyl-2-methyl-1,3-dioxolane (μg)	2.09 (4750)^b^		0.044
1-acetyloxy-2-propanone (μg)		12.2 (132)^b^	9.23
2-monoacetin (μg)		46.8 (156)^b^	30
1,2,3-propanetriol, diacetate (diacetin) (μg)	1.23 (323)^b^		0.381
1,4-dioxane, 2-ethyl-5-methyl (μg)	0.055 (13750)^b^		0.0004
12,14-labdadiene-7,8-diol, (8a,12E) (μg)	1.43 (2234)^b^		0.064
1-hydroxy-2-butanone (μg)	0.947 (204)^b^		0.465
1-hydroxy-2-propanone(1,2-propenediol) (μg)	162 (167)^b^	1135 (226)^b^	96.8–502
2 (5*H*)-furanone (μg)	5.32 (267)^b^		1.99
2*H*-pyran-2-one, tetrahydro-5-hydroxy (μg)		8.16 (196)^b^	4.13
2,3-dihydro-5-hydroxy-6-methyl-4*H*-pyran-4-one (μg)	0.231 (171)^b^		0.135
2-cyclopentene-1,4-dione (μg)	3.8 (497)^b^	8.4 (418)^b^	0.764–2.01
2,4-dimethylcyclopent-4-ene-1,3-dione (μg)	0.333 (173)^b^		0.193
2(5*H*)-furanone (μg)		5.45 (256)^b^	2.13
2-furanmethanol (μg)	39.2 (560)^b^	37.5 (396)^b^	7–9.47
2-furanmethanol, 5-methyl (μg)	0.123 (424)^b^		0.029
2*H*-pyran-2-one, tetrahydro-5-hydroxy (μg)	4.45 (143)^b^		3.11
2-methylcyclobutane-1,3-dione (μg)	2.78 (392)^b^		0.71
2-propanone, 1-(acetyloxy) (μg)	16.9 (211)^b^		8.01
3 (2*H*)-furanone, dihydro-2-methyl (μg)	0.326 (274)^b^		0.119
5-methylfurfural (μg)	0.995 (157)^b^	14.2 (270)^b^	0.632–5.25
anhydro linalool oxide (μg)	0.457 (157)^b^		0.291
benzenemethanol, 4-hydroxy (μg)	0.011		not detected
butyrolactone (μg)	4.08 (560)^b^	4.8 (444)^b^	0.728–1.08
cyclohexane, 1,2-dioxo (μg)	0.083 (180)^b^		0.046
cyclohexane-1,2-dione, 3-methyl (μg)	0.101 (138)^b^		0.073
ethyl 2,4-dioxohexanoate (μg)	6.73 (189)^b^		3.57
isolinderanolide (μg)	4.99 (270)^b^		1.85
methyl furoate (μg)	0.147 (507)^b^		0.029
pyranone (μg)	6.54 (129)^b^	51.4 (116)^b^	5.07–44.5
pyranone (μg)	9.26 (159)^b^		5.84

a The results compare the yields from an IQOS stick with the reference 3R4F cigarette, however, on an equivalent tobacco basis, the IQOS yields should be multiplied by at least 3.2. HCI, Health Canada Intense smoking regimen.

b Compound in IQOS aerosol as a percentage of amount in 3R4F reference cigarette smoke.

## Indoor Environments

The chemical constituents in side stream emissions from IQOS have been investigated to assess their suitability for indoor environments. PMI researchers, Mitova et al.,^54^ found that the indoor concentrations of nicotine, acetaldehyde, and glycerine from IQOS aerosol were above background level but significantly below the harmful levels defined in air quality guidelines, concluding that the use of IQOS in an indoor environment with adequate ventilation does not adversely affect the overall indoor quality. Another PMI study (Meisutovic-Akhtarieva et al.^32^) observed that the use of IQOS results in significant increase of several analytes from its emissions including nicotine, acetaldehyde and particulate matter concentration within indoor air. The authors suggested that the intensive use of IQOS in a confined space with limited ventilation might cause substantially elevated concentrations of volatile harmful species, such as acetaldehyde. The independent study by Cancelada et al.^55^ observed high level of acrolein and several other harmful compounds in the side stream emissions of IQOS and concluded that although IQOS is a weaker indoor pollution source than conventional cigarette its impact is not negligible. Indeed, significant levels of *n*-alkanes, organic acids and carcinogenic aldehydes including formaldehyde, acetaldehyde, acrolein have also been observed in IQOS side stream aerosol,^3^ suggesting that the use of IQOS should be restricted in indoor environments even though the concentrations of these compounds are lower in IQOS aerosol compared to CC smoke.^3^

## Can PAHs Form by Thermal Breakdown in IQOS?

The release of PAHs and certain phenols,^8^ and levoglucosan^3^ (1,6-anhydro-β-glucopyranose) that are markers of pyrolysis and combustion of biomass^49,56,57^ in IQOS emissions^3,8^ have raised questions about the temperatures reached in the IQOS device. PAHs, in particular are products of high temperature pyrolysis of tobacco and biomass only forming in appreciable amounts above 500 °C,^46,49,58^ much higher than the maximum temperature of 320–350 °C reported for IQOS.^4,6,8,29,38^ Cozzani et al.^4^ and other PMI researchers^38^ attributed the presence of benzo[a] pyrene in IQOS emission to the contamination of tobacco leaves from the environment (resulting from other combustion sources) during growing and curing, because PAHs do not occur naturally in biomass. However, comparing the background PAHs of IQOS tobacco for a recent PMI study^59^ to PAHs in IQOS emission from an earlier PMI study^38^ (Table 5) for the same sets of samples suggests this is too simplistic an explanation and that PAH formation via pyrolysis could be occurring in IQOS, potentially at hot spots.

**Table 5:** Comparison of Background PAHs of IQOS (THS 2.2) Tobacco Stick to the Deliveries in the Emissions and 3R4F Reference Cigarette Smoke^a^

	Goujon et al.^59^ (Supporting Information Table S9)			Schaller et al.^38^ (Supporting Information Tables I and II)		
	IQOS tobacco background PAH (ng/stick)			deliveries in emission/smoke HCI (ng of stick/ng of 3R4F cigarette)		
sample	benzo[a]pyrene	pyrene	benzo[a]anthracene	benzo[a]pyrene	pyrene	benzo[a]anthracene
FR1	16.6	82.1	22.5	1.02	8.01	2.64
A	118.8	1381.7	347	9.10	185.65	45.66
B	56.9	243	91.2	4.34	25.26	13.33
C	<0.06	12.5	<0.05	0.35	4.50	1.02
D	48.4	568.7	138.9	3.97	63.30	20.52
E	<0.06	18.1	<0.05	0.55	4.71	1.09
3R4F				15.0	79.3	27.2

a FRI: THS 2.2 regular blend of air cured, bright flue-cured and aromatic oriental (sun-cured) tobaccos, same as FR1 monitor blend in Schaller et al.^38^ A: Aromatic fire-cured tobacco, same as AR1 in Schaller et al.^38^ B: Flue-cured bright tobacco, same as FC5 in Schaller et al.^38^ C: Flue-cured bright tobacco, same as FC6 in Schaller et al.^38^ D – Blend of flue-cured and aromatic fire cured tobaccos, same as BL1 in Schaller et al.^38^ E: Blend of flue-cured and aromatic oriental sun-cured tobaccos, same as BL11 in Schaller et al.^38^ 3R4F: University of Kentucky reference cigarette, blend of flue-cured, air-cured (Burley and Maryland), oriental (sun-cured) and reconstituted tobaccos. HCI: Health Canada intense smoking regimen. PAHs:Polycyclic aromatic hydrocarbons. Data from both studies were from Supporting Information except 3R4F data, which was in main article of Schaller et al.^38^

Table 5 compares the background PAHs in an IQOS heat stick tobacco^59^ to those released in IQOS (THS 2.2) emissions using various tobacco blends (FR1, A–E, see Table 5 for description of the different tobacco blends) in heat sticks and the 3R4F reference cigarette.^38^ The yields of PAHs released are lower than heat stick background levels for tobaccos with high yields of background PAHs (samples FR1, A, B and D), on one hand suggesting they may arise primarily from distillation. However, for the two tobacco blends with very low yields of background PAHs (samples C and E), the concentrations of benzo[a]pyrene (BaP) and benzo[a]anthracene (BaA) are higher in IQOS emission than the background levels suggesting also that PAHs are being formed via pyrolysis. For sample C, BaP increased from 0.06 (background level) to 0.35 ng (IQOS delivery) and BaA increased from 0.05 (background level) to 1.02 ng (IQOS delivery). The authors might argue that the yield of BaP in IQOS delivery for sample C was below the limit of quantification, and thus the value given was that for the limit of quantification. However, the yield of BaA in IQOS delivery (sample C) was quantified. Similarly, for sample E, BaP and BaA increased from 0.06 and 0.05 ng, respectively (background level also given as the value for the limit of quantification), to 0.55 (BaP) and 1.09 ng (BaA), which were actual yields in IQOS emission that were quantifiable.

For the 3R4F reference cigarette with a blend similar to FR1 tobacco (Table 5), BaP and pyrene in the smoke are less than the background levels for FR1 tobacco, while the yield of BaA in the smoke is ∼20% higher than the background level. This suggests that a significant proportion of PAHs in CCs smoke might be from the background PAHs in tobacco and not from combustion as currently suggested by almost all studies. Overall, for IQOS, these results suggest that, albeit in relatively small quantities, the increase in concentration of PAHs in IQOS emissions compared to initial tobacco PAHs background levels may indicate a contribution from pyrolysis. These would suggest that hot spots could exist to reach temperatures significantly higher than the bulk maximum temperatures reported in the range of 320–350 °C discussed earlier.^4,6,8,29,38^

## Discussion and Recommendations for Further Research

Albeit still containing the same HPHCs as released in CC smoke, the previous sections have highlighted that IQOS emissions, in terms of their temperature of release, they do fit the definition of smoke containing compounds, such as levoglucosan^3^ that are markers of biomass combustion^56,57^ and black carbon^3^ that are associated with biomass, wood, and tobacco smoke.^33,34^ The previous discussion has identified key knowledge gaps that need to be addressed, the need to compare HPHCs yield of IQOS to CCs on a tobacco basis and the uncertainties concerning the increased emission levels that occur from continual reheating and the maximum temperatures reached in IQOS. We now summarize how these gaps can be tackled.

As already highlighted, the continual reheating of the deposited tar and char in the IQOS device is likely to result in the generation of higher concentrations of HPHCs and particulate matter than from single use. Therefore, analysis after repeated use needs to be investigated to provide more reliable assessments of the compounds released from IQOS in relation to human use as recommended by the manufacturer before cleaning the device. Further, it is essential to measure the fraction of tar or NFDPM that is insoluble black carbon to provide further evidence that IQOS emissions can be classed as smoke and, also, to do this comparison on a mass of tobacco basis.

### The Need for In-Depth Characterization

The chemical evidence to date indicates that IQOS generates HPHCs and other compounds that are a cause for concern regarding human health. There is a clear need to measure the whole range of compounds released from HTPs as a basis for understanding potential health effects. However, the overall characterization of the compounds in IQOS emissions is thus far limited compared to assessments of CC smoke, where over 5000 individual compounds species have been identified^60^ compared to just 529 in HTPs.^52^

Bentley et al.^52^ in this PMI study using a combination of gas and high-pressure liquid chromatography with high resolution mass spectrometry, have identified over 500 compounds released from IQOS at concentrations greater than 100 ng per heat stick, but all of these were also present in CC smoke. The authors claim that these account for over 95% of the tar excluding nicotine, but as described earlier (Figure 2), this tar yield was a factor of 3 lower than in earlier studies meaning that the 95% of the tar accounted by the 529 compounds could be a vastly overestimate. Further, many of the compounds identified by St. Helen et al.^26^ as being released in greater concentrations from IQOS than CC were not reported by Bentley et al.^52^

Although soft ionization methods that give only parent ions without any fragmentation in mass spectrometry clearly does not resolve isomers where front-end chromatographic separation is a necessity, this approach is nevertheless useful since the elemental formulas of every species present can be obtained. For example, in petroleum, 8000 compositionally distinct species have been observed by single electrospray ionization coupled with high resolution Fourier transform ion cyclotron resonance mass spectrometry.^61^ This would be extremely useful baseline for matching individual compounds where gas or high-pressure liquid chromatography are first required to separate isomeric species prior to high resolution mass spectrometry.

The fact the HTPs are subjected to lower temperatures and undergo a lesser degree of thermal decomposition regarding the number of chemical bonds cleaved suggests the number of species at concentrations similar or greater than 1 ng per stick will be vastly higher than the 529 compounds reported by Bentley et al.^52^ Such a low threshold needs to be used so that all HPHCs, such as the full suite of PAHs, will be included, given that no safe level of exposure to cigarette smoke exist not even second-hand cigarette smoke.^62^ More in-depth compositional information, in conjunction with more independent clinical research, will better equip regulators to assess the health risks posed by IQOS and other HTPs.

### Pyrolytic Formation and Release of Compounds

To resolve the issue for compounds already present in tobacco in measurable concentrations, particularly PAHs that arise from curing and probably to a lesser extent, transportation, emissions from normal and pre-extracted tobacco need to be compared to quantify the formation and release of all the compounds released from IQOS arising as a direct result of pyrolysis. Such studies could also involve spiking tobacco with ^13^C isotopically labeled compounds, which would provide a detailed picture of all the reactions they potentially mediate through following the fate of the ^13^C label.

### What Are the Maximum Temperatures Reached?

Further, to answer the question as to whether uneven temperature profiles in heat sticks can lead to potential hot spots that could, for example, lead to PAH formation, pyrolysis studies on tobacco and other constituents of HTPs need to be performed together with more effort on heating tobacco blends under controlled temperature/time conditions. Such studies have considered PAHs^49^ and phenols^63^ at high temperatures in relation to CC smoke, but more focus is needed on lower temperatures, in the case of IQOS starting at the window of 200–350 °C, below and within the maximum temperatures so far quoted for IQOS heat sticks. This will provide more information on the evolution of the species identified (Table 4) that evolve in higher concentration from IQOS than CC smoke. Further, specific compound ratios will provide temperature proxies. For example, the ratio of alkylated PAHs to the corresponding unsubstituted PAHs, 2/3 ring to 4/5 ring PAHs and monohydric (e.g., phenol and cresols) to dihydric phenols (e.g., catechol) all increase with temperature. Regarding phenols, the study by McGrath et al.^63^ indicates that dihydric phenols are formed from tobacco mainly above 350 °C, the maximum temperature reported for IQOS heat sticks. Further light on the pyrolytic origin of PAHs, phenols and other compound classes can be obtained from isotopic labeling as mentioned previously but also form normal ^13^C compound specific measurements where from PAHs, differences will exist between extraneous PAHs arising from curing and transportation and the PAHs formed by thermal breakdown.

## Conclusions

The yields of harmful and potentially harmful constituents (HPHCs) from IQOS tobacco sticks in relation to conventional cigarettes need to be multiplied by a factor of 3.2–3.6 if a comparison is made on a tobacco basis. The HPHCs present are the same as in conventional cigarette (CC) smoke, albeit in lower concentrations and formed at lower temperatures, analogous to the emissions from the earlier generation of HTPs, which were classed as smoke. Also, IQOS emissions contain carbon particles with most of the compounds released being formed by chemical reactions provides further evidence that IQOS emissions fit the definition of being both an aerosol and a smoke. Continual reheating of deposited tar in the IQOS device will occur with real-life use and is likely to result in the generation of higher concentrations of HPHCs and particulate matter. Despite the evidence that IQOS heats to no more than 350 °C, there is uncertainty over the maximum temperatures reached in heat sticks and local hot spots could cause the formation and release of species, such as phenol/cresols and polycyclic aromatic compounds (PAHs), typically not formed in significant amounts by the thermal breakdown of tobacco and biomass until much higher temperatures.

Regarding the need for further research on IQOS emissions, the impact of repeated use to determine the levels of black carbon (insoluble organic matter) in the particulate matter, and the extent to which compounds in IQOS emissions are formed by pyrolysis need to be assessed rigorously. To address whether uneven temperature profiles in heat sticks can lead to potential hot spots that could, for example, lead to PAH formation, pyrolysis studies on tobacco and other constituents of HTPs are required in conjunction with more effort on heating tobacco blends under controlled temperature/time conditions.
